# Centralized project-specific metadata platforms: toolkit provides new perspectives on open data management within multi-institution and multidisciplinary research projects

**DOI:** 10.1186/s13104-022-05996-3

**Published:** 2022-03-18

**Authors:** Andrew Wright Child, Jennifer Hinds, Lucas Sheneman, Sven Buerki

**Affiliations:** 1 Institute for Interdisciplinary Data Science, Research Computing and Data Services, University of Idaho, Moscow, ID 83844-2358 USA; 2 Department of Biological Sciences, Boise State University, Boise, ID 83725-1515 USA

**Keywords:** Data management, Open data, Metadata, Multi-institutional, Multidisciplinary, Data science, Toolkit

## Abstract

Open science and open data within scholarly research programs are growing both in popularity and by requirement from grant funding agencies and journal publishers. A central component of open data management, especially on collaborative, multidisciplinary, and multi-institutional science projects, is documentation of complete and accurate metadata, workflow, and source code in addition to access to raw data and data products to uphold FAIR (Findable, Accessible, Interoperable, Reusable) principles. Although best practice in data/metadata management is to use established internationally accepted metadata schemata, many of these standards are discipline-specific making it difficult to catalog multidisciplinary data and data products in a way that is easily findable and accessible. Consequently, scattered and incompatible metadata records create a barrier to scientific innovation, as researchers are burdened to find and link multidisciplinary datasets. One possible solution to increase data findability, accessibility, interoperability, reproducibility, and integrity within multi-institutional and interdisciplinary projects is a centralized and integrated data management platform. Overall, this type of interoperable framework supports reproducible open science and its dissemination to various stakeholders and the public in a FAIR manner by providing direct access to raw data and linking protocols, metadata and supporting workflow materials.

## Introduction

Open data (OD) is growing in popularity among scientists and encourages research reproducibility and integrity. Within the scientific community OD is expanding by both funder and publisher requirement [1–4]. Furthermore, scientific research is under increased scrutiny, as studies are failing to stand the test of reproducibility and integrity, and public distrust increases due to misinformation and political agendas [5–9]. Subsequently, new guidance from Open Science initiatives such as TOP guidelines and FAIR (Findable, Accessible, Interoperable, Reusable) principles prioritize the ideals of transparent open research [10–15]. Thus, although some researchers have hesitations about sharing research data and protocols, the use of OD is increasing as studies have demonstrated that OD create an inclusive environment for junior researchers, increases research recognition and credibility, inspires new research ideas, and has the potential to reduce costs of future research [1–4, 16–18]. Furthermore, open access (OA) to raw and processed data as well as source code and sufficient documentation of research protocols promotes FAIR principles and maximizes reproducibility [8, 19].

A critical component of OD and open science is the documentation of complete and accurate metadata, workflow, and code which all increase likelihood of reproducible and replicable results. However with the massive scope and seemingly endless choices of metadata schemes, it can be challenging to determine which metadata scheme is “best” or even preferred to record, store, reference and share metadata and workflow associated with published OD products [20]. It is a best practice in data/metadata management to use established and internationally accepted metadata formats (e.g., DDI, ISO 19115, ABCD, Darwin Core, EML, dbEST). Although standardized metadata schemata provide uniform metadata collection formats, they frequently are designed for a single discipline, and adaptations for multidisciplinary collaborations, although possible, can create obstacles for researchers with limited metadata training.

Some researchers have endeavored to increase open science by funding the publication of OA peer-reviewed manuscripts, and have supported FAIR principles by  also providing OA to raw data, protocols, project workflow and code using less formalized metadata formats (e.g., integrated workflow utilizing the R Markdown language coupled with GitHub) [21–24]. Documenting metadata and project workflow with these newer flexible formats provides the ability to document multidisciplinary research and package project metadata within a less formalized structure. However, this approach lacks an expressive metadata format that promotes findability and reusability, and the resources to consistently and accurately catalog OD products because they do not utilize standardized metadata schema. Thus, the process to accurately document and catalog metadata within multidisciplinary collaborative research projects such as large-scale research awards (e.g., NSF EPSCoR, NSF LTER, USDA NIFA, NIH, etc.,) can be complicated.

For example, consider multidisciplinary research aimed to tackle pressing research related to assessing the effect of climate change on natural habitats/ecosystems (such as the GEM3 project discussed in this paper). Such integrated research requires implementing frameworks to support data sharing, integration, and transparency to foster innovative solutions to complex problems [25, 26]. Pioneering research initiatives or toolkits that strive to bridge cross-institutional and multidisciplinary research collaborations can aid researchers as they address complex environmental and societal issues by providing platforms to integrate high-impact transparent research that can be shared FAIRly and publicly.

We suggest and demonstrate that one solution to increase data FAIRness and elevate research integrity within multi-institutional and multidisciplinary research projects is a centralized, customized, and project-specific data management platform built using interoperable components.

## Main text

As part of a five-year NSF EPSCoR Track 1 grant (OIA-1757324) aimed at discovering fundamental knowledge of genetic mechanisms that can predict how organisms adapt to changing environments and thus inform evidence-based management of natural resources, we developed an interactive data/metadata platform where researchers can provide OA to metadata, raw data, and data products with research collaborators, externally affiliated organizations, and the public. The GEM3 project focuses on two keystone species (i.e., Big Mountain Sagebrush and Redband Trout) of the US Intermountain West ecosystems that are pivotal to regional socio-ecological systems. This project applies a multidisciplinary approach to unravel the mechanisms underpinning adaptive capacity of populations to ultimately model and predict ecosystem trajectories in the face of climate change and regional population growth. The project combines multidisciplinary research strengths in bioinformatics, complex modeling, ecology, fisheries science, genomics, geospatial science, remote sensing, and social-ecological systems science to contribute to one of the most compelling and contemporary national challenges of our time – understanding the *Rules of Life* [27, 28]. In addition to promoting FAIR principles, the project also embarks on a transformative approach at answering complex questions by integrating the strengths of multidisciplinary research teams to address complex environmental and societal issues [29]. To facilitate data and metadata sharing across the diversity of disciplines represented in the project, we developed a centralized research dashboard (https://www.idahogem3.org/data-dashboard).

The dashboard is a web-based toolkit providing an interconnected inclusive environment acknowledging the specific needs of each discipline yet establishing a unified structure and ontology connecting each discipline within the project (e.g., project-wide controlled vocabulary, data/metadata standardization initiatives). Due to the complex relationships and breadth of metadata that naturally exist within multidisciplinary and multi-institutional research projects, design of the dashboard required guidance from an inclusive working group comprised of data managers, web-developers, and selected representatives from all participating Idaho research universities (i.e., Boise State University, Idaho State University, University of Idaho) chosen to represent the diversity of GEM3-specific components and research fields. The group also serves as a conduit to promote usage of the toolkit and provide feedback from researchers to improve, expand and maintain capabilities. This ensures that there is a clear link between the technical implementation team and researchers to ensure long-term usage and functionality.

Centralized web-based research dashboards can be constructed using open-source software and packages (e.g., Drupal, WordPress, Python/Flask, etc.,), as were the tools described in this paper (i.e., Drupal). Regardless of the software/platform of choice, we propose that data/metadata from multidisciplinary collaborative projects can be cataloged using discovery-level metadata, while a modular, interoperable design provides the ability to link, attach, and export standardized discipline-specific metadata. This design decreases user confusion from solely using jargon-heavy and discipline-specific metadata forms and improves searchability across multiple disciplines during data/metadata queries by unifying discovery-level metadata fields across all project disciplines. Additionally, the design allows best practices in data/metadata management to persist within records (e.g., controlled vocabularies, attachment standardized metadata schemes, standardized date formats, version control) [30].

**Fig. 1:**
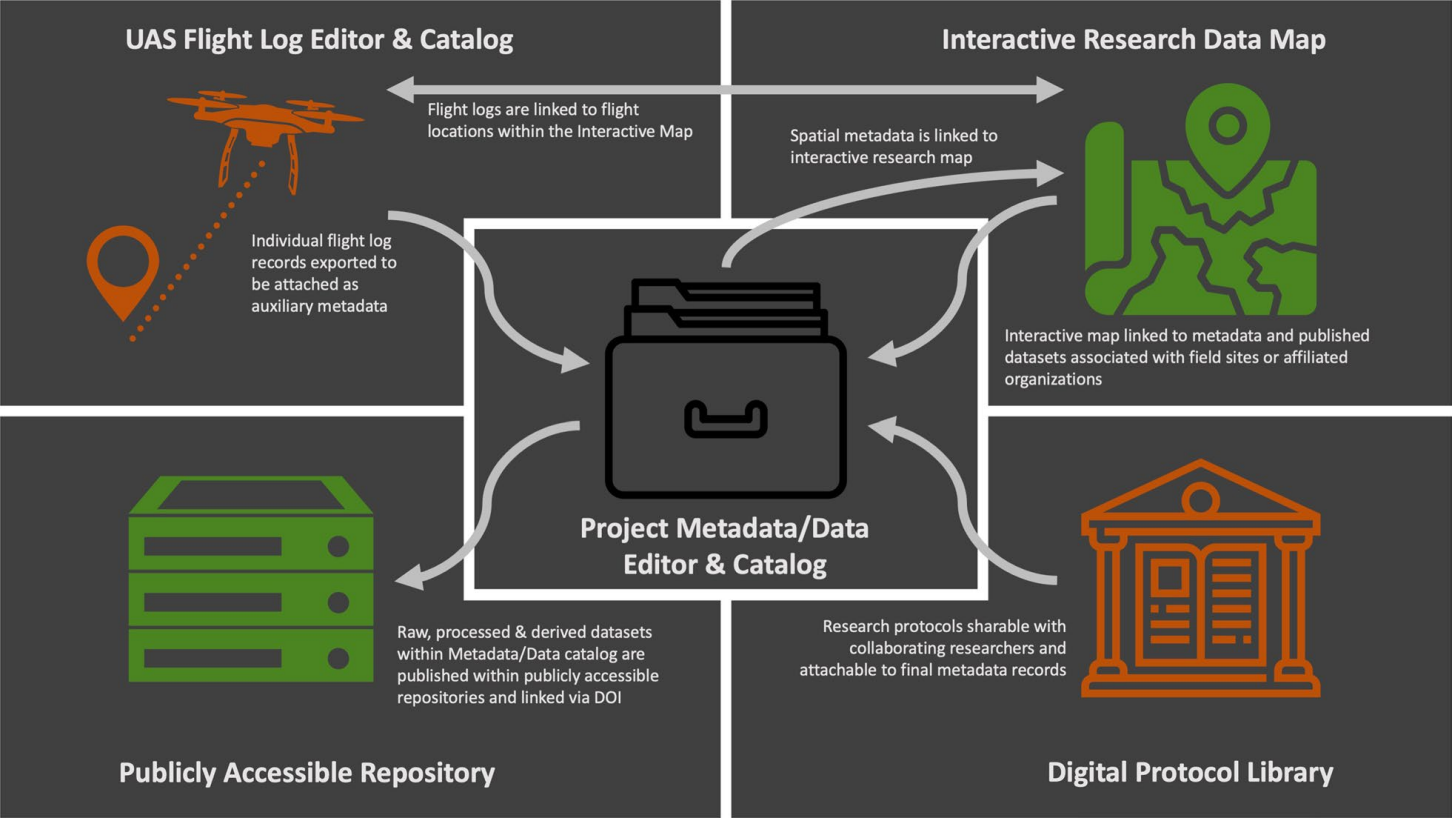
Centralized metadata/data platform design and functionality schematic. Icon Credits: kareemov1000, Nawicon, lastspark, Eucalyp and Andy Miranda from the Noun Project (https://thenounproject.com/)

The centralized dashboard platform described in this paper contains three main components: (1) an interactive map, (2) protocol library, and (3) metadata editor/catalog; and the inclusion of a project-specific unoccupied aerial systems (UAS) flight log editor/catalog module (Fig. 1).

### Interactive map

In addition to storing site and project-specific metadata for active and legacy research sites, the Interactive Map (https://www.idahogem3.org/data-map) links people, metadata, OD, research protocols and UAS flight missions to both research sites and externally affiliated organizations on a publicly searchable spatial platform. The map contains features to query site/project metadata based on keywords, research interests, project components, data collected, and whether the research is transdisciplinary [31]. Additionally, it allows users to compare the spatial proximity of cross-disciplinary sites, overlay the boundaries of social-ecological and UAS study areas in relationship to field sites, visualize the historic extent of organisms of interest, and envision relationships between research lands and the territories of indigenous peoples that originally inhabited areas of interest [32]. The interactive map is a publicly accessible and practical visual tool designed to foster collaborations, provide links to OD, link spatial metadata with data records and UAS flight logs and identify possible gaps in collected data (Fig. 1).

### Protocol library

The protocol library (https://www.idahogem3.org/protocols) was designed to provide capabilities to share and update research and data acquisition protocols in an online environment that was protected from public access and provided version control documentation. The library restricts public permission to either submit protocols or access protocol files, until they are attached and publicly released with a published OD record (Fig. 1). This ensures that researchers can freely share emerging research protocols across multi-institutional or multidisciplinary collaboration agreements and eventually authorize public visibility of these protocols within metadata records, while also protecting against early unauthorized release.

### Metadata/data editor and catalog

The metadata editor webform follows a modular design approach, which requires researchers to submit common discovery metadata (e.g., title, authors/creators, spatiotemporal extents, keywords, OECD subject/discipline, funding, DOI), while also allowing attachment of embedded or associated discipline-specific standardized metadata files based on user selections within the webform [30]. Additionally, the metadata webform is crosslinked to the interactive data map, so records can be spatially searched based on the location of data acquisition; and crosslinked with the protocol library to allow the attachment of research protocols to final metadata records. Although permission to access the metadata editor is restricted to project participants, all metadata records and associated OD within those records are publicly searchable within a metadata/data catalog.

The metadata/data catalog (https://www.idahogem3.org/metadata-catalog) provides OA to metadata and data records, and faceted search options to query records based on keywords, authors, OECD discipline/subject, project component, year completed, data availability, and externally affiliated organizations [33]. The catalog has the capabilities to link metadata with published datasets using DOIs issued from popular OD repositories (e.g., Dryad, NCBI, Figshare, Harvard Dataverse, Open Science Framework, Zenodo). Additionally, it can link records to customized workflow that may include protocols, code, scripts, and/or data stored in less popular sources (e.g., institutional repositories, personal GitHub repositories) and other related content (e.g., peer reviewed scholarly publications, related datasets, websites) by URL or DOI. Due to the dynamic nature of files stored within GitHub repositories, it is imperative that catalog records point to static releases of content versions used within a specific study [34]. Furthermore, submission of static releases of datasets to persistent OD repositories (e.g., Zenodo, Dryad, Figshare, institutional repositories) is recommended over storing these records within personal GitHub repositories. The metadata/data catalog is a publicly accessible central catalog designed to encourage findability and accessibility of all data records regardless of discipline and connect published OD from multiple repositories with essential metadata, workflow, and code to encourage interoperability and reproducibility during reuse (Fig. 1).

### UAS flight log editor and catalog

The UAS flight log editor and catalog (https://www.idahogem3.org/uas-flight-logger-search) are not an essential component of the centralized metadata/data platform, but it demonstrates how project-specific tools can be designed within these frameworks to create, catalog, and link customized auxiliary metadata with OD records. Like previously discussed tools, public access to the flight log editor is restricted, and only authorized participants can submit flight records. These records are automatically linked to locations on the interactive map, and once finalized, can be exported for attachment as auxiliary metadata within an OD record. The UAS flight log catalog is accessible to the public; however, access to data files and flight details are restricted, and only selected metadata fields are visible (i.e., mission datetime, mission location, drone and sensor selections, sensor bands recorded, and principal investigator contact information) until the data creators release flight details within OD records. The UAS flight log editor and catalog are an example of a specialized auxiliary metadata collection module that can feed discipline-specific metadata into final OD records upon publication, while also providing storage and sharing capabilities for project collaborations (Fig. 1).

## Outlook

In addition to fostering research integrity, transparency, and reproducibility by abiding FAIR principles, our unique centralized metadata platform attempts to bridge the gap of discipline specific research silos by providing OA to metadata, protocols, and associated OD and OD products within an integrated and searchable project-specific platform. For example, the sagebrush biome is one of the most imperiled ecosystems of North America, with more than 350 plant and animal species of conservation concern within its bounds and occupying less than 55% of its original extent [35]. Researchers from multiple disciplines (e.g., genetics/genomics, botany, ecology, climatology, geography, sociology), scales (e.g., gene, species, subspecies, community, ecotone, ecosystem, landscape), and institutions (e.g., research universities, PUIs, government agencies, Tribal governments, NGOs) are all investigating possible mechanisms and solutions to help conserve this delicate ecosystem while accounting for population growth. Therefore, this type of centralized metadata/data platform could become a multidisciplinary reference implementation for researchers working within the sagebrush biome or other equally complex multidisciplinary environments. Overall, this type of interoperable framework supports reproducible Open Science and its dissemination to various stakeholders and the public in a FAIR manner by providing OA to raw data while also providing access to protocols, metadata and supporting workflow materials.

## Data Availability

Data sharing is not applicable to this article as no datasets were generated or analyzed during the current study. Access to the tools described in the paper are publicly accessible at https://www.idahogem3.org/data-dashboard.

## Abbreviations

ABCD: Access to biological collection data
dbEST: Expressed sequence tag database
DDI: Data documentation initiative
DOI: Digital object identifier
EML: Ecological metadata language
EPSCoR: Established program to stimulate competitive research
GEM3: Genes to environment: modeling, mechanisms and mapping
LTER: Long-term ecological research
NCBI: National center for biotechnology information
NGO: Non-governmental organizations
NIFA: National Institute of Food and Agriculture
NIH: National Institute of Health
NSF: United States National Science Foundations
OD: Open data
OA: Open access
OECD: Organization for Economic Co-operation and Development
PUI: Primarily undergraduate institution
TOP: Transparency and openness promotion
UAS: Unoccupied aerial systems
URL: Uniform resource locators
USDA: United States Department of Agriculture
